# The Challenge of Die Filling in Rotary Presses—A Systematic Study of Material Properties and Process Parameters

**DOI:** 10.3390/pharmaceutics12030248

**Published:** 2020-03-10

**Authors:** Ann Kathrin Schomberg, Arno Kwade, Jan Henrik Finke

**Affiliations:** 1 Institute for Particle Technology, Technische Universität Braunschweig, Volkmaroder Str.5, 38104 Braunschweig, Germany; a.kwade@tu-braunschweig.de (A.K.); j.finke@tu-braunschweig.de (J.H.F.); 2 Center of Pharmaceutical Engineering (PVZ), Technische Universität Braunschweig, Franz-Liszt-Str. 35A, 38106 Braunschweig, Germany

**Keywords:** tableting, die filling, rotary tablet press, process understanding, paddle feeder, turret speed, paddle speed, filling efficiency, kinetic filling limitations, densification

## Abstract

For the efficient and safe production of pharmaceutical tablets, a deep process understanding is of high importance. An essential process step during tableting is the die filling, as it is responsible for a consistent tablet weight and drug content. Furthermore, it affects the results of subsequent process steps, compaction and ejection, and thus critical quality attributes. This study focuses on understanding the influences of process parameters and material properties on die filling on a rotary tablet press. By the systematic variation in process parameters as the turret and paddle speeds as well as the fill and dosing depths, five formulations with differing properties are processed. Analysis of the normalized tablet weight, called filling yield, revealed different limitation mechanisms of the filling process, i.e., incomplete filled dies for certain parameter settings. Kinetic limitations occur due to a short residence time under the feed frame (filling time) caused by high turret speeds, which additionally induce high tablet weight variation coefficients. Characteristic maximum turret speeds at certain paddle speeds can be found to still achieve complete filling. At low turret speeds, densification of the powder inside the dies takes place, induced by two mechanisms: either high paddle speeds or high overfill ratios, or a combination of both. The challenge to fill the dies completely as well as avoid densification is dependent on material properties as the flowability. The mass discharge rate from an orifice was found to be in a linear correlation to the filling results of different formulations below complete filling.

## 1. Introduction

In the pharmaceutical industry, deeper process understanding is gaining importance. This is emphasized by the Quality by Design (QbD) approach promoted by health authorities [1]. In the context of continuous manufacturing, which is an aspirational aim, process understanding, among others enabling a reliable process control, has even a greater priority. The present study focuses on the systematic understanding of the die filling process directly in rotary tablet presses. The primary objective of this work is to qualitatively describe the influence of the process parameters and material properties on the die filling of selected model materials. Derived knowledge can be used to enable the development of process models to forecast quantitative impacts, and thus shorten the development time of novel products and increase process stability.

The tableting process can be divided into four steps: powder feeding, die filling, compaction and ejection. The product quality fundamentally depends on die filling. It critically affects the tablet quality, as weight variations contribute to drug content variations. Furthermore, subsequent process steps such as compaction and ejection depend on the result of filling. If die filling is not accurate, the effective pressure acting on the powder bed, applied by the opposing punch movement, undercuts the processing limits. Thus, quality attributes like tensile strength, porosity and dissolution behavior can be influenced. To meet the required quality, it is necessary to ensure a complete, reproducible and consistent die filling.

Different mechanisms have been identified for die filling, which involve *gravity filling*, *suction filling* and *forced feeding* [2]. *Gravity filling* occurs for powder flowing into a die cavity under gravity [3]. *Suction filling* takes place due to an arising pressure gradient from the downward movement of the lower punch under the powder bed inside the feed frame, pulling the powder into the die cavity. Furthermore, improvement in the filling process is achieved by feeding systems with moved components, referred to as *forced feeding*.

Industrial rotary tablet presses commonly use a forced feeding system consisting of a feed frame equipped with one to three stirrers, called *paddle feeder*. In the most common two-wheel setup, the first wheel, called the feeding wheel, delivers powder into the die while the second wheel, the metering wheel, transfers the exceeded powder back into the feed frame. In some constructions, a third wheel is positioned in the center above the feeding and metering wheel to supply powder equally to both wheels [4].

The dosing depth results from the fill depth, which is defined by the fill cam, minus the overfilling height, which is used to support complete die filling [5]. Thus, after previous overfilling, the lower punch moves upwards to eject some powder for weight control. The mechanisms, including *forced feeding*, *suction filling* and *overfilling* as well as disruptive factors like centrifugal forces, vibrations and vertical stress caused by the above powder, acting simultaneously in a rotary tablet press, result in a complex process [6]. Variable parameters, including turret speed, paddle speed, fill depth and dosing depth, help to control the process. In addition, powder properties clearly affect the filling quality and efficiency.

Several studies were carried out to understand the filling process using a linear filling device [2,3,7,8,9,10,11,12]. Wu et al. examined the flow of metallurgical powders in stationary simple and stepped dies from a moving feeding shoe in air and vacuum [3]. It was found that powder properties, shoe speed and airflow affect the filling process. Sinka et al. also used a linear filling device with *gravity fill* in air and vacuum to investigate the flow properties of pharmaceutical powders to try to resemble die filling on a rotary press [12]. The critical velocity, which still allows completely filled dies, was used for classifying the flow properties. The strong improvement in the filling result due to *suction fill*, which can take place in a rotary press, was detected by Jackson et al. [2]. Besides the experimental investigations, CFD/DEM-Simulations focused on the die filling process and analyzed air effects [13,14,15,16,17,18,19,20,21,22,23,24]. A rotational filling device with *gravity fill* equipped with moved dies and a stationary feeder, mimicking a rotary press more realistically, was used to correlate die filling performance and critical material attributes [25]. Furthermore, rotational systems with forced feeding were selected to investigate the powder residence time and the impact of feed frame speed and die speed on the die filling [26,27]. With increasing feed frame speed, the separated powder mass was increasing, while an increase in die speed led to a decrease in powder mass. In addition, the flowability of lubricated powders was improved due to overlubrication caused by increasing feed frame speed. In addition to the approaches with filling devices, computer modeling was used to investigate the flow behavior of powders in feed frames and into dies [19,20,21,22].

Although there were several research studies describing the filling efficiency, these simplified systems are able to investigate effects of selected parameters, but often important parameters were not taken into account, which is why the general transfer of findings is limited [5]. There are only few studies carried out on rotary tablet presses. The impact of flow behavior of powders on die filling properties was examined by Yaginuma and a PLS model for thirty blends, predicting tablet weight variability, was developed by van Snick, but the application of their findings on other materials and tablet presses is limited [28,29]. Other studies focus on specific issues like feed frame design and filling level inside a feed frame by laser triangulation and in-line NIR spectroscopy [4,30,31,32,33]. General relations between powder properties, operating conditions and tablet weight and its variability are rare.

In contrast, Peeters et al. investigated the influence of turret speed, paddle speed, fill depth and material properties with a Design of Experiments (DoE) by using microcrystalline cellulose (MCC) and dibasic calcium phosphate dihydrate (DCP) directly on the rotary tablet press MODULE™ P (GEA) [5]. The interaction between the process parameters and both the tablet weight and weight variability were examined, identifying the turret speed and the paddle speed of the feeding wheel as strong impact factors. In addition, the weight variability of MCC is affected by the paddle speed of the metering wheel. Contrasting the powders, the impact of the paddle speeds on tablet weight is more pronounced for MCC due to its fairly flowing behavior. On basis of the DoE, the optimum combinations of process parameters are presented to meet requirements for minimum tablet weight variability. However, these determined optimum parameters are singular solutions for the investigated powders in combination with the used rotary press. Accordingly, further advancement would be provided by developing general and transferable deductions from such datasets instead.

Due to the lack of deep understanding, the aim of the current work was to systematically investigate the influence of particular process parameters and powder properties to achieve an improved understanding of the mechanistic relations regarding tablet weight and weight variation. This is performed directly on a rotary press to avoid differences in operating modes that may finally hinder the transferability of models from testing rigs to industrially relevant machines and processes. The findings should also be transferable to other powders and formulations.

## 2. Materials and Methods

### 2.1. Materials

Microcrystalline cellulose (MCC, Vivapur^®^ 102, JRS Pharma, Rosenberg, Germany) and anhydrous dicalcium phosphate (DCPA, Emcompress^®^ Anhydrous, JRS Pharma, Rosenberg, Germany) were used as model materials. Magnesium stearate (MgSt, Magnesia GmbH, Lüneburg, Germany) was used as a lubricant. The characteristic particle sizes of MCC and DCPA are in a comparable range with slightly lower particle sizes for MCC (Table 1). The solid, bulk and tapped density of DCPA is higher, almost double, than MCC.

### 2.2. Methods

#### 2.2.1. Powder Characterization

Particle size distribution was determined by laser light diffraction (Mastersizer 3000, Malvern Panalytical, Kassel, Germany). Three measurements were performed, and the mean values were calculated. The solid densities of the powders were measured in triplicate with the helium pycnometer Ultrapyc 1200e (Quantachrome Instruments, Boynton Beach, FL, USA). The bulk and tapped density were determined according to the Ph. Eur. 9.3 2.9.34 using a 100 mL cylinder, tapped by a volumetric analyzer (Erich Tschacher Laboratoriumsbedarf, Bielefeld, Germany). Measurements for each sample were carried out in triplicate, calculating the average. Based on the bulk and tapped density, the Hausner ratio *H* and Compressibility index *C* were calculated as follows

(1) $$H=\frac{\rho_{t}}{\rho_{b}}$$

(2) $$C=\frac{\rho_{t}-\rho_{b}}{\rho_{t}}\times 100$$

Flow properties were determined using a granulate flow tester (ERWEKA GmbH, Langen, Germany) by measuring the time required for 100 g of the powder sample to flow through an orifice of 25 mm according to the Ph. Eur. 9.3 2.9.16. The orifice has an opening angle of 40° and is made of stainless steel. From these results, the powders’ mass flow rate was calculated. Furthermore, the ring shear tester RST-XS (Dr. Ing. Dietmar Schulze Schüttgutmesstechnik, Wolfenbüttel, Germany) was used to determine flow properties. Therefore, preshear stresses of 1, 2, 4 and 8 kPa were used with corresponding shear to failure stresses of 20, 50 and 80% (33% for 1 kPa). On basis of the consolidation stress σ_1_ and the unconfined yield strength σ_c_, the *ff_c_* is calculated as follows [34]

(3) $$ff_{c}=\frac{\sigma_{1}}{\sigma_{c}}$$

#### 2.2.2. Powder Blending

The three blends of MCC and DCPA were prepared using a cube mixer (ERWEKA GmbH, Langen, Germany). The volume of 3.5 L was filled up to 40%. Initially, DCPA was mixed with 1 wt % magnesium stearate (MgSt) for five minutes at 30 rpm, then MCC was added and mixed for another ten minutes. The composition of the three blends processed on the XL 100 are displayed in Table 2.

#### 2.2.3. Die Filling/Tableting Experiments

Die filling/tableting experiments were carried out with the rotary tablet press XL 100 (KORSCH AG, Berlin, Germany) in lab scale. Therefore, tablets were produced at different process parameters, determining their weight afterwards as a measure for the filling weight (*n* = 10) (Quintix^®^224 – 1CEU, Sartorius, Göttingen, Germany). For the investigation, a paddle feeder with one wheel—the feeding wheel—was used. The press was equipped with four flat-faced round Euro-D punches with a diameter of 9 mm. No external vacuum cleaner for aspiration was used. In order to investigate the influence of different fill depths, three fill cams with fill depth of 10, 14 and 16 mm were used; the corresponding dosing depth and operating speeds can be found in Table 3. For each fill depth a minimum and a maximum dosing depth, resulting from a machine-specific maximum overfill of 5.5 mm as well as a minimum overfill of 0.5 mm, were chosen. To enable the comparison of the different fill depths at the same dosing depth, intermediate dosing depths corresponding to the minimum dosing depths of the neighboring larger fill cam were applied. Low dosing depths were selected to evaluate possible densification effects, while high dosing depths were chosen to investigate whether complete filling could be achieved. For all experiments, three turret speeds as well as four paddle speeds were used, including the machine-specific minimum and maximum as well as intermediates. In order to gain more detailed information at the highest dosing depth, representing the highest challenge of filling, turret speeds of 30 and 50 rpm are additionally investigated.

## 3. Theoretical Background

### 3.1. Definition of Terms

**Figure 1:**
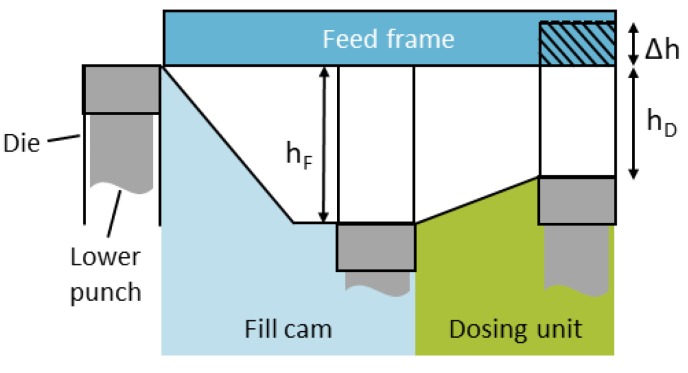
Schematic overview of the punch positions used for filling with perspective from the side – fill depth *h_F_*, overfill *Δh* and the resulting dosing depth *h_D_*.

In the following, important terms are named, and equations, used for the evaluation below, are defined. Under the feed frame, the lower punch is moved down by the fill cam to a certain position, called *fill depth h_F_* (Figure 1). To change the *fill depth*, another fill cam, a stationary mechanical component, must be installed. As described above, the die cavity is overfilled, thus, for dosing, the lower punch is lifted. The height, by which the punch is moved upwards until scraping off the powder bed at the end of the feed frame, is named *overfill Δh*. Therefore, the so-called *dosing depth h_D_* is reached and the filling process is completed by scraping powder above the die (Figure 1). The remaining powder inside the die is compacted afterwards.

Different *dosing depths* can be achieved by stepless variable height adjustment. Correspondingly, *overfill* can vary in the range of 0.5 to 5.5 mm for each *fill depth* set. The kinematic volume reduction during this process is described by the *overfill ratio r_o_*, defined as the ratio of *overfill* to *fill depth* (Equation (4)).

(4) $$r_{o}=\frac{\Delta h}{h_{F}}$$

Regarding different *dosing depths* as well as powders with different bulk and tapped densities, normalization is necessary to allow the comparison of the resulted tablet weight as the dimensionless result of filling. Therefore, the *filling yield φ* is defined as the ratio of the tablet weight *m_T_* to the theoretical maximum weight resulting from the dosing volume inside the die, calculated with the die radius *r_die_* and the *dosing depth h_D_*, as well as the tapped density *ρ_t_* of the respective powder (Equation (5)).

(5) $$\varphi =\frac{m_{T}}{\pi \cdot r_{die}^{2}\cdot h_{D}\cdot \rho_{t}}$$

The tapped density is used due to the assumption that maximum powder consolidation in the feed frame and during die filling is comparable to the impact of the taps, which leads to the greatest change in particle packing in normal direction due to the powder weight. For this reason, it is hypothesized that the tapped density can be used as a limit for consolidation inside the die without compaction, and accordingly the maximum filling yield amounts to 1.

Corresponding to the consolidated state, a reference value for complete die filling in the unconsolidated state, related to the bulk density, can be defined. Therefore, the filling yield represents complete die filling for the ratio of the weight resulting from the dosing volume filled with bulk density *ρ_b_* to the weight resulting from the dosing volume filled with tapped density, called *unconsolidated complete die filling UCF* (Equation (6)). The equation can be simplified to the ratio of bulk density to tapped density which equals the inverse of the Hausner ratio.

(6) $$UCF=\frac{\pi \cdot r_{Mat}^{2}\cdot h_{D}\cdot \rho_{b}}{\pi \cdot r_{Mat}^{2}\cdot h_{D}\cdot \rho_{t}}=\frac{\rho_{b}}{\rho_{t}}<1$$

The variation in the filling yield is described by the *tablet weight variation coefficient WVC* which equals the relative standard deviation (Equation (7)). It is calculated as the percentage of the standard deviation of the mean value of the filling yield.

(7) $$WVC=\frac{\sqrt{\frac{1}{n-1}\sum_{i=1}^{n}{\left(\varphi_{i}-\bar{\varphi }\right)}^{2}}}{\bar{\varphi }}\times 100$$

### 3.2. Detailed Process Description

**Figure 2:**
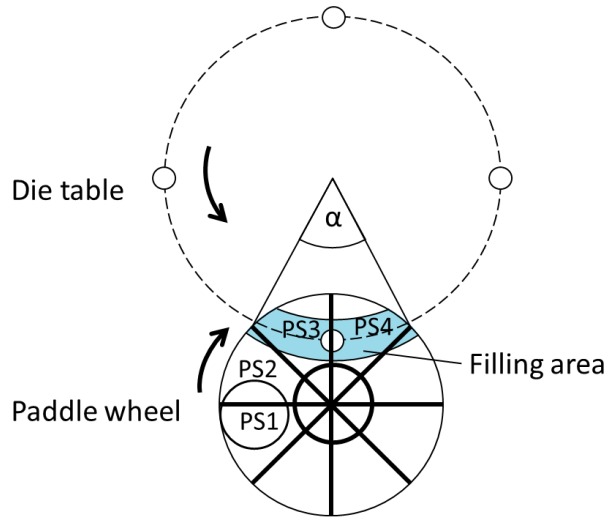
Schematic overview of the four process steps for die filling in a one-paddle feed frame. The paddle rotates clockwise, the die table rotates counter-clockwise.

For a better understanding and explanations of the following experimental data, the filling process is described in detail. Die filling can be divided into four process steps. The area in which the respective process step takes place is marked with the abbreviations in a schematic overview with plan view (Figure 2):

PS1—powder feeding from the hopper into the feed frame;
PS2—powder transport by the feeding wheel (into the filling area);
PS3—die filling;
PS4—dosing out: reduction of die fill volume (by Δh).

During PS1, the powder flows from the feeding system, a hopper, into the feed frame. Here, it enters the intersections between the paddles of the paddle wheel. The mass flow from the hopper determines the filling level of the interspaces in combination with the removal mass flow by the dies. Methods to measure the filling level are developed but not used under continuous production conditions yet [30,31,32]. Hypotheses about the filling level and the linked flow mechanisms, which are proposed to explain the powder flow, should be evaluated with these measurements in the future. For poor flowing powders, the intersections might not be filled with as much powder as for free-flowing powders. This could be a limiting factor for successful filling at a high turret speed. However, the diameter of the pipe entering the feed frame is large, so a sufficient powder volume flow is likely unless the bridging of highly cohesive powders occurs.

**Figure 3:**
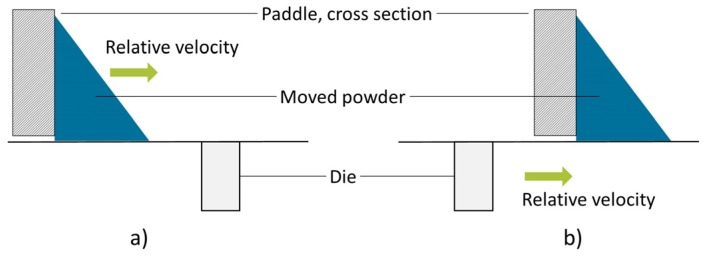
Schematic representation of the two possible states during die filling (PS3): (**a**) paddles overtake dies, (**b**) dies overtake paddles in the filling area. The green arrows represent the relative velocity.

The following process step, PS2, describes the powder movement induced by the paddle wheel. As Dühlmeyer et al. show in their work, the powder is not homogenously distributed over the radius as well as over the width of an interspaces [30,31,32]. With increasing paddle speed, the powder is moved radially towards the outer wall of the filling chamber and the filling area (Figure 2, light blue) due to centrifugal forces as a function of the paddle speed squared. This means that the powder, which is initially on an inner radius, might enter the filling area and therefore supply new powder which can be filled into the dies. Dühlmeyer et al. present that, for a fixed powder mass inside the feed frame, the powder accumulates in front of the pushing paddles. Therefore, the powder bed develops a bulk geometry with a triangular vertical cross area along the circumference (Figure 3). Mateo-Ortiz et al. also observed a wave-like powder flow inside the feed frame due to the paddle movement [4]. This may influence the powder flow into the die. A change in bulk density due to possible densification of the powder has not yet been investigated. For a compressible powder, such a consolidation would lead to worse flow behavior for subsequent die filling.

**Figure 4:**
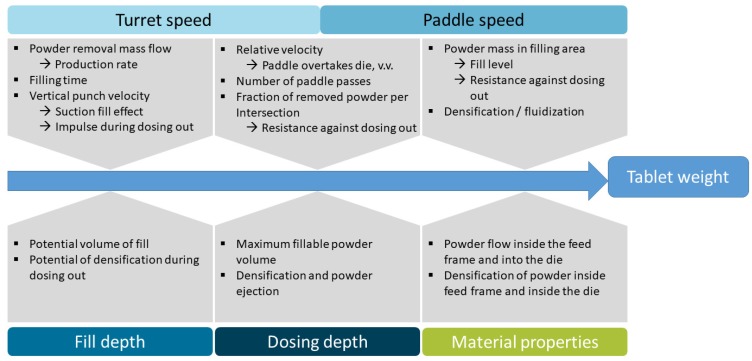
Summarized influences of process parameters and material properties on the tablet weight during die filling.

Following the powder transport, the die is filled in PS3. The residence time under the feed frame is one influencing parameter, which is set by the turret speed and the angle of the filling area, marked as α (Figure 2). With increasing turret speed, the period of time shortens, which affects the results of die filling (Figure 4). In parallel, a higher turret speed creates a higher pressure drop for suction filling due to the increased vertical punch velocity (Figure 4). For experimental investigations using the rotary tablet press XL 100, a feed frame with an installed external lubrication of the die was used, which implies that the lower punch is moved downwards before entering the feed frame. Therefore, the suction fill effect might be small or even negligible. The combination of the process parameters turret and paddle speed has a strong influence on the die filling. Regarding the rotational speeds of the paddle and the turret, two states for die filling can be distinguished—either the paddles overtake the dies in the filling area, represented by a positive relative velocity (Figure 3a), or the dies pass the paddles if the relative velocity is negative (Figure 3b). On basis of the hypothesis that the powder is accumulated and probably densified in front of the pushing paddles, more powder would enter the die if the paddles push the powder over the die (Figure 3a). Wu et al. investigated the powder flow from a linear moved fill shoe into static dies and found a landslide-like flow of the front region for low relative velocities, named nose flow, which supports complete die filling [3]. In the feed frame, nose flow can develop if the interspaces are not filled completely with powder and if the relative speed between the powder and the die is low enough and positive. Otherwise, especially if the die overtakes the paddles with powder in front, bulk and intermittent flow for poor flowing powders may occur [3]. If a suction pressure develops, which is unlikely in the used experimental setup, as described, it has a significant influence on the powder flow. Besides the different flow mechanisms, the absolute value of the relative velocity between the paddle, moving the powder, and the die might be a crucial factor. A low relative velocity correlates with a longer period of time for the particles to flow into the die without the shift of a paddle over the die.

After die filling, the filled die volume is reduced in PS4 by a partial upwards movement of the lower punch in the dosing out phase. The acceleration of the powder mass inside the die results in particle rearrangement, and thus, in the reduction of porosity and, possibly, the ejection of excessive powder (Figure 4). In dependence on the fill level of the feed frame, ejecting powder experiences a resistance due to overhead powder. For high fill levels inside the feed frame, the resistance could lead to densification of the powder inside the die, resulting in densities above the bulk density ρ_b_. Therefore, filling yields between 1 and the *UCF* can be reached. Possibly, for high fill levels, the powder is already densified inside the feed frame, and thus during die filling. However, it is currently not possible to quantify the mass inside the die and its apparent bulk density before ejection of the tablet. Accordingly, insights into the initial state of the powder inside the die can only indirectly be deduced from systematic studies.

The different impacts on the tablet weight as the result of die filling, which are described in this section, are displayed in the summarized representation (Figure 4).

## 4. Results and Discussion

### 4.1. Powder Characteristics—Flow Behavior

**Figure 5:**
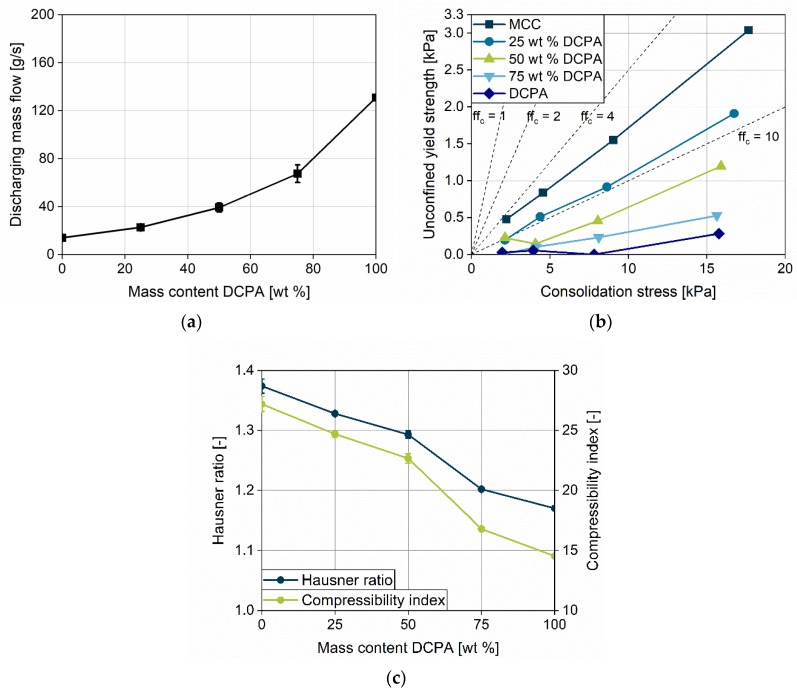
Flow properties of the powders used in this study; (**a**) Mass flow of powders, measured by granular flow tester; (**b**) Flow function (*ff_c_*) measured with ring shear tester; (**c**) Hausner ratio and Compressibility index for the five formulations used.

Since the flow property of a powder is a crucial factor in the context of die filling, different techniques for its determination were used. In the granular flow tester, the mass flow was determined for all powders used in this study (Figure 5a). The progressive rise in the mass flow shows a constant improvement in the flow property with increasing DCPA mass fraction. Thus, this method indicates that DCPA mass flow is more than four times greater than that of MCC. The flow functions of the powders used in the study were calculated using a ring shear tester (Figure 5b). Great differences can be found for the pure substances DCPA and MCC. DCPA shows very high values of *ff_c_* in the range of >20, so flow properties are excellent. Contrasting this, the *ff_c_* for MCC is comparably low, between 4 and 6, which indicates poor flow properties, still classified as *easy-flowing* [34]. The *ff_c_* for blends of the two powders are systematically located between the pure substances. The order corresponds to the mass fractions of DCPA and MCC. Therefore, the blends represent the gradation in the flow properties determined by both tests applied.

The Hausner ratio and the Compressibility index of the powders allow for an estimation of the materials’ compressibility (Figure 5c). MCC has a high compressibility, which is almost double the value of DCPA. The blends show a compressibility between the pure substances, although it does not decline linearly. Blends below a mass content of 75 wt % DCPA possess a considerably higher compressibility and Hausner ratio compared to powders with higher content.

### 4.2. Impact of Process Parameters on Filling Yield

The impact of different process parameters such as the turret speed, the paddle speed, the filling and dosing depth as well as the overfill are investigated in the following. A limited set of experiments is highlighted to point out the impact of each parameter variation. Due to the greatest challenge of filling, the highest fill depth of 16 mm and the highest dosing depth of 15.5 mm were chosen in most cases to display the strongest and most fundamental effects.

The turret and paddle speed, as the dynamic parameters of the system, simultaneously influence the die filling by their interaction. The turret speed is directly linked to the tablet production rate (Figure 4), which is desired to be as high as possible due to economic reasons. The maximum applicable speed is technically determined by the rotary press on one hand and by quality requirements for the tablets on the other hand. The maximum tablet production rate which can safely meet the requirements depends on the formulations properties and the combination of the chosen values of the static process parameters (h_F_, h_D_, ∆h) and the paddle speed. Thus, in the following, general understanding of the impact and interplay of each process parameter is investigated based on the most extreme formulations of MCC and DCPA. As the blends show results between the pure substances, they are not displayed separately in this section. The influence of the material properties of the blends is investigated and discussed in Section 4.3. There, the filling yield of all formulations used is displayed, using dependence on the paddle speed as an example.

#### 4.2.1. Impact of Turret Speed on the Filling Yield

**Figure 6:**
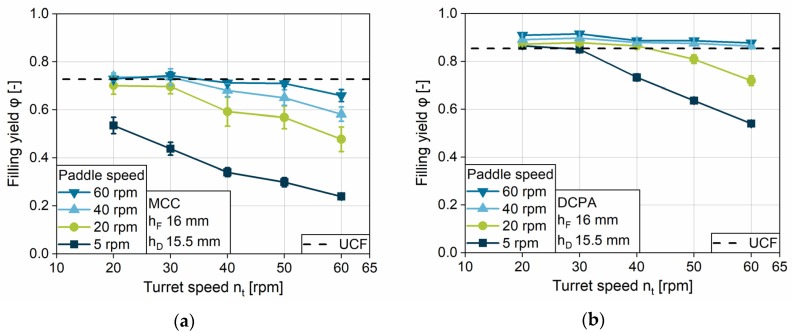
Impact of the turret speed on the filling yield for (**a**) MCC and (**b**) DCPA at different paddle speeds at a fill depth of 16 mm and a dosing depth of 15.5 mm.

The impact of the turret speed on the filling yield is analyzed at five rotational velocities for four different paddle speeds. In addition to the data, the graphs show the *unconsolidated complete die filling* (*UCF*) in order to compare the actual filling yield with the complete filling at an unconsolidated state. For MCC, one can observe that only at low turret speeds of 20 and 30 rpm, and in parallel high paddle speeds of 40 and 60 rpm, *UCF* is achieved (Figure 6a). With increasing turret velocity, the filling yield of MCC shows a continuous decrease in all paddle speeds. DCPA achieves complete die filling at every turret speed if paddle speeds of 40 and 60 rpm are applied, while the filling yield decreases as well for 20 and 5 rpm paddle speed (Figure 6b). The considerably lower filling yields for MCC in comparison to DCPA at the same turret und paddle speed can be related to the different flow properties. The initial powder flow into the die for MCC might be considerably lower compared to DCPA and might not develop properly in the short period of time. The hypothesis is supported by the lower mass flow of MCC, investigated with the granulate flow tester (Figure 5a). In addition, regarding the previous process steps PS1 and PS2, less powder might be transported by the paddles and by centrifugal forces for MCC into the filling area as compared to DCPA at a given paddle speed. Nonetheless, the fill level in the filling area was not directly measureable.

Comparing the progression of the data with increasing turret speed, one can observe a maximum turret speed up to which point the dies can be filled completely at a certain paddle speed for each material. Above the maximum turret speed in a first approximation, the filling yield shows a linear decline. The maximum turret speed for *UCF* as well as the slope may be interpreted as characteristic values influenced by the paddle speed and the powder properties. As described for PS3, for higher turret speeds the die remains for a shorter period of time under the feed frame. For this reason, the challenge of complete die filling is magnified with increasing turret speed, especially for powders with poor flow properties. Coincident observations related to the decline can be found in the literature [5,28]. Peeters et al. presented different linear gradients for the tablet weight with increasing turret speed at two different paddle speeds [5]. However, no further general findings and correlations were derived.

The strong impact of the paddle speed can be observed by comparing the different rotational speeds of the feeding wheel. The higher the paddle speed, the higher the maximum turret speed at which complete filling can generally be achieved. Further investigations on the impact of paddle speed on the filling yield are discussed in the following section.

The decline in the filling yield is based on the discussed reasons for incomplete filling referred to as a kinetic limitation of the system, which is a general finding for the process. The start of the limitation, the maximum turret speed to achieve *UCF*, depends on the material properties on one hand, as well as on the process parameters on the other hand.

#### 4.2.2. Impact of Paddle Speed on the Filling Yield

**Figure 7:**
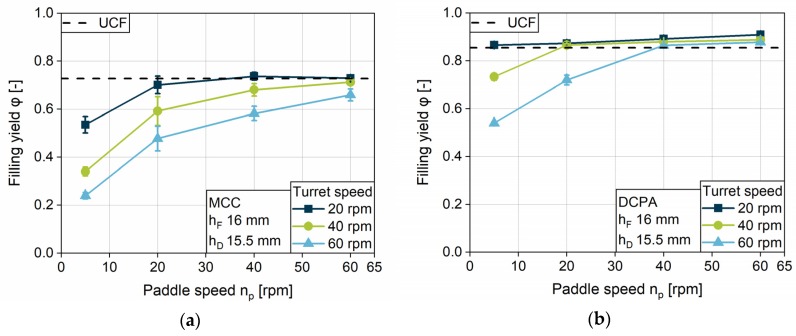
Impact of the paddle speed on the filling yield for (**a**) MCC and (**b**) DCPA with varying turret speed.

In order to better clarify the influence of the paddle speed, the filling yields achieved at a fill depth of 16 mm and a dosing depth of 15.5 mm, as the highest filling challenge, are presented for MCC (Figure 7a) and DCPA (Figure 7b) as a function of the paddle speed. To show the impact of combination to the turret speed, three speeds, including the slowest, middle and the fastest, are chosen. The paddle speed shows a consistent influence for all experiments and powders—the filling yield increases in a degressive way with increasing paddle speed until achieving *UCF*. Similar observations were made by Mendez et al. [26]. The slope and, as already discussed in Section 4.2.1, the paddle speed needed to achieve complete die filling are dependent on the material and its flow properties. While the gradient for MCC is high at the beginning, due to the low filling yields at 5 rpm paddle speed, it flattens with increasing paddle speed. In contrast, DCPA shows a lower gradient at low paddle speeds, which declines only slightly. With increasing paddle speed, more powder is delivered to the filling area in a certain time (PS2), which can fill the dies. Additionally, a higher paddle speed reduces the relative speed between the dies and the powder moved in the feed frame, as described for process step PS3 (Figure 4). Furthermore, the increased induced kinetic energy might support the powder flow into the dies.

As the graphs for DCPA show filling yields above *UCF*, consolidation inside the feed frame and the die can occur and increase as a function of paddle speed under the same static conditions (h_F_, h_D_, ∆h). On the contrary, at the parameter setting displayed, MCC does not show densification above *UCF* but asymptotically reaches the *UCF*, indicating that no consolidation by dynamic parameters was achieved for MCC for this case.

In general, one can find a minimum paddle speed at a certain turret speed to overcome the kinetic limitation and to fill the dies completely. The higher the turret speed and the poorer the flow properties of the material, the higher the minimum paddle speed needs to be. An increasing paddle speed has a considerably higher impact on the filling yield of fairly flowing (MCC) compared to free-flowing powders (DCPA).

#### 4.2.3. Impact of Turret Speed and Paddle Speed on the Tablet Weight Variation Coefficient

**Figure 8:**
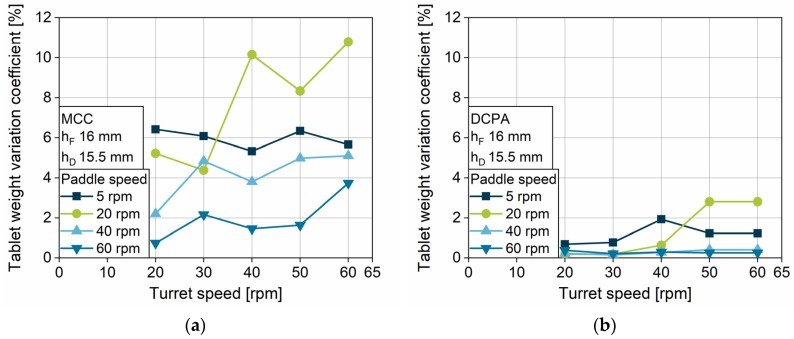
Effect of turret speed on the tablet weight variation for (**a**) MCC and (**b**) DCPA at different paddle speeds.

Besides the tablet weight, which is determined by the filling yield, the *tablet weight variation coefficient* (*WVC*) is a crucial factor for tablet production. It is important to gain reproducible tablet weights to ensure constant drug content. The *WVC*s of MCC and DCPA show strong differences as function of the turret and paddle speeds (Figure 8a,b). As Yaginuma and Bellini also presented, the *WVC* for both, MCC and DCPA, increases with rising turret speed [26,33]. The free-flowing DCPA has very low *WVC*s for paddle speeds of 60 and 40 rpm, while for 20 and 5 rpm paddle speed the value grows with increasing turret speed. Comparing the data to the *WVCs* of MCC, it becomes clear that the weight variation in MCC tablets is about four times higher than for tablets consisting of DCPA, due to the differing flow properties. The *WVCs* for 20, 40 and 60 rpm rise with increasing turret speed, while the *WVC* for 5 rpm is approximately constant on a high level. Unexpectedly, for both powders, the variation coefficient for 20 rpm exceeds the lower paddle speed and achieves values of about 11% for MCC (Figure 8a) and 3% for DCPA (Figure 8b) at high turret speeds.

In order to clarify this phenomenon, it is necessary to better differentiate the limitation that is causing the variation in general. With higher turret speeds, the kinetic limitation leads to decreasing filled powder masses. In case the filling yield is considerably below the *UCF*, which means the kinetic limit of complete filling is widely exceeded, the amount of filled powder is so low that only the volume of air inside the die is reduced by dosing out. Therefore, the resulting powder mass only depends on the process step of die filling. If the kinetic limitation is reached, the filled powder mass leads to a filling yield below *UCF*. However, the powder height inside the die may be between the filling and dosing depth, while the apparent density is below the bulk density. In this case, powder ejection and densification can occur simultaneously during dosing out. Here, the resulting filling yield depends on two process steps, die filling and dosing out. This may hypothetically lead to higher *WVC*s in comparison to the filling yield, which only depends on the filling.

**Figure 9:**
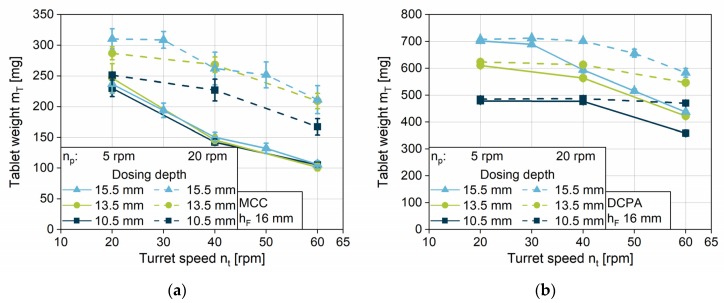
Effect of turret speed, paddle speed and dosing depth on the relative tablet weight variation for (**a**) MCC and (**b**) DCPA at different paddle speeds.

These two phenomena can be identified by comparing the total tablet weight for different dosing depths. For MCC, tablet weight at 5 rpm paddle speed falls on the same masses for all dosing depths (Figure 9a). For DCPA, this phenomenon can only be found under the limiting combination of 5 rpm paddle speed, turret speeds of 40 rpm and above, and dosing depths of 15.5 and 13.5 mm (Figure 9b). In these cases, based on the hypothesis above, no powder is ejected during dosing out. Contrasting this, the tablet weights, especially for DCPA, decrease at 20 rpm paddle speed but differ for the various dosing depths, which leads to the assumption that powder is still dosed out. These findings contribute to the explanation for the *WVC* for a paddle speed of 20 rpm exceeding those of 5 rpm provided above.

#### 4.2.4. Impact of the Fill Depth

**Figure 10:**
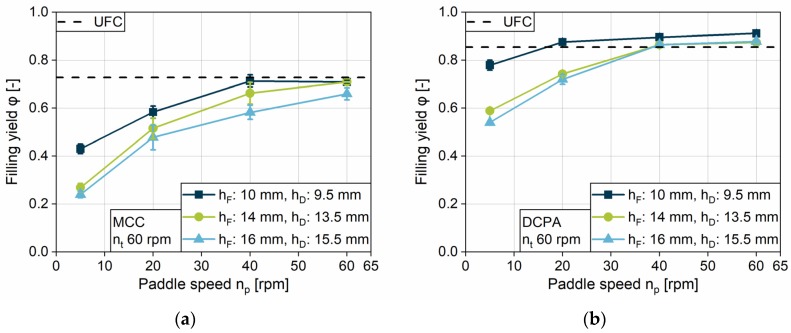
Filling yield for different fill depths for (**a**) MCC and (**b**) DCPA.

The filling yield is investigated for the three different fill depths: 10, 14 and 16 mm. To yield comparable data, the linked minimum overfill of 0.5 mm and the highest turret speed of 60 rpm were chosen, respectively. For both formulations, MCC and DCPA, the higher the fill depth, the lower the filling yield at a certain turret speed (Figure 10a,b). With increasing fill depth, the challenge for die filling increases and the relative filling yield decreases. For MCC, the difference between the filling yield at 9.5 mm dosing depth in comparison to deeper ones is higher at a low paddle speed of 5 rpm compared to an increased speed. The free-flowing DCPA presents considerably low differences for 13.5 and 15.5 mm dosing depths, especially in contrast to the lowest filling, i.e., a dosing depth of 9.5 mm. DCPA achieves better results for die filling in comparison to MCC, especially at a high fill depth, owing to their differences in flowability. In addition, for MCC the paddle speed needs to be higher to overcome the kinetic limitation and to reach *UCF* in combination with the increasing fill depth. Contrasting the results for MCC, DCPA, unusually, achieves *UCF* for the dosing depth of 13.5 and 15.5 mm at the same paddle speed. Although the overfill is low, at 0.5 mm each, densification inside the dies induced by the increased paddle speed takes place for DCPA.

In general, the increased die volume due to a higher fill depth leads to higher required paddle speeds to achieve the same filling yield as for lower volumes. Besides a higher transport rate of the powder, increased centrifugal forces result in a higher powder mass in the filling area, which might support the filling. Further investigations are needed in order to take influences as the fill level inside the feed frame into account. This may enable the identification of possible proportionalities.

#### 4.2.5. Impact of the Dosing Depth

**Figure 11:**
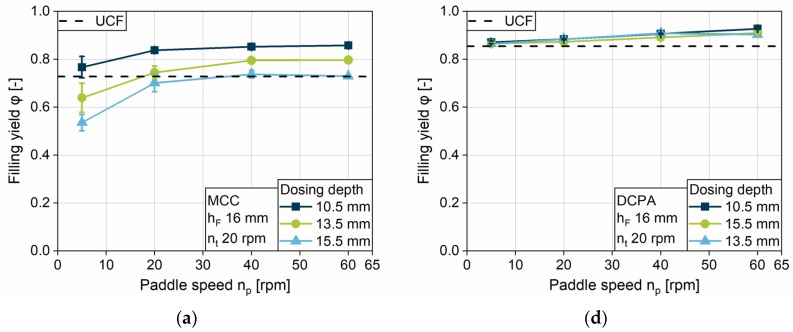

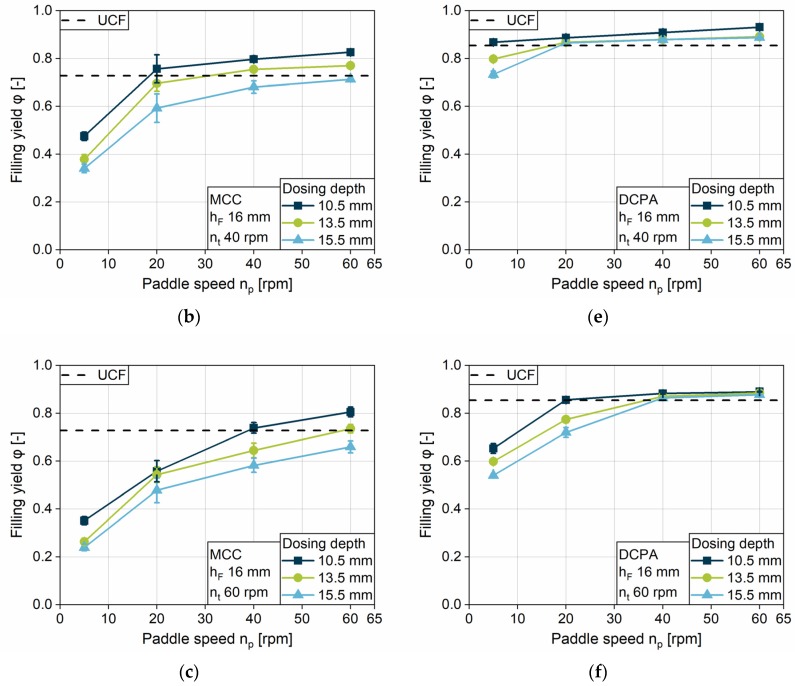
Filling yield for different dosing depth at a fill depth of 16 mm at 20, 40 and 60 rpm turret speed for MCC (**a**,**b**,**c**) and DCPA (**d**,**e**,**f**).

Following the section of filling, dosing takes place by an upward movement of the lower punch. Therefore, the filling yield is influenced by this process step, and by the dosing depth used. The filling yield of three different dosing depths (10.5, 13.5 and 15.5 mm) at a fill depth of 16 mm are presented for MCC (Figure 11a,b,c) and DCPA (Figure 11d,e,f) for three turret speeds. For MCC and DCPA, it can be observed that the dosing depth has a considerable influence on the filling yield. Independent of the turret speed, the lowest dosing depth of 10.5 mm performs best in die filling, based on the lower final volume after dosing out, which has to be filled with powder. Due to the normalization of this volume, filling yields are higher. In addition, lower dosing depths lead to a densification inside the die for both powders, MCC and DCPA. It is observable that the densification above the *UCF*, which is caused by the overfill, is considerably lower for DCPA than for MCC. The difference can be explained by the varying compression behavior of the powders. The increasing amount of powder, which needs to be dosed out, leads to a considerably stronger increase in density of the bulk inside the die for a compressible powder (MCC, i.e., high Hausner ratio, see Figure 5). The mechanisms are described in Section 3.2 for process step PS4. Densification by dosing out is characterized by a parallel shift in the filling yield to higher values, applicable for filling yields above *UCF*.

In addition to the densification by dosing out, filling yields above *UCF* rise with increasing paddle speed. For MCC at 20 rpm turret speed (Figure 11a), the change in density of the bulk is strong above the *UCF* and levels off for high paddle speeds at a filling yield of about 0.85. At lower turret speeds, the increase in the filling yield is almost constant. However, the filling yields are still below 0.85, which might be due to lower acting stresses on the powder at the certain process parameters. Less time for consolidation due to the shorter filling time might also be a contributing factor. In contrast, the filling yield for DCPA rises linearly with increasing paddle speed over the whole range studied. However, this effect, and the slope of the filling yield above *UCF*, decreases with higher turret speeds.

In general, higher dosing depths require higher paddle speeds to fill the dies completely. With increasing turret speed, the paddle speed which ensures complete filling and induces densification rises as well. One can observe different behaviors for the change in density of the bulk, depending on the dosing depth and the paddle speed. Furthermore, it is noticeable that both powders do not reach the tapped density, according to a value of 1 for the filling yield at the displayed parameter settings. The stress, applied neither by paddle force feeding, dosing out, nor the combination of both, is high enough at the given process parameters.

#### 4.2.6. Impact of Overfill

**Figure 12:**
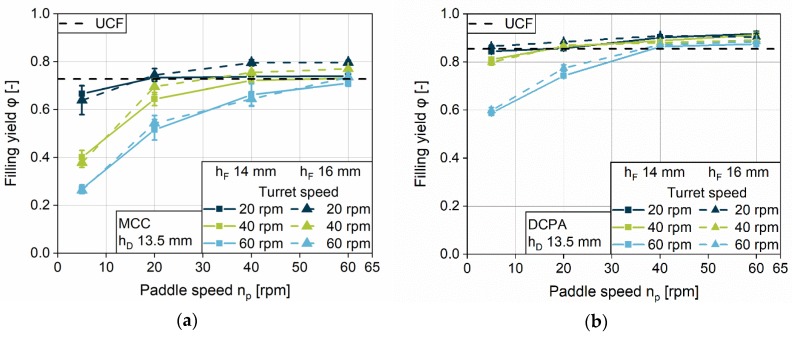
Impact of the paddle speed on the filling yield for (**a**) MCC and (**b**) DCPA with varying turret speed.

In order to elucidate the influence of the overfill, the filling yield for MCC (Figure 12a) and DCPA (Figure 12b) is displayed for a fill depth of 14 and 16 mm, both resulting in a dosing depth of 13.5 mm. Comparing the filling yield below *UCF*, no considerable difference between the two fill depths is observed for both powders. The filled powder mass is normalized to the same volume for both datasets—the dosing volume. Thus, the data show that the filled powder mass is independent of the fill depth as long as the filling is below the *UCF*. In this case, no powder is ejected during dosing out. For 14 mm fill depth, the dies are filled completely at a certain paddle speed, but no densification is observable for MCC. Contrasting this, for 16 mm fill depth, filling yields above the *UCF* are observable. Therefore, the apparent density inside the die after dosing out is above the bulk density. The higher filling volume at 16 mm enables more powder to enter the die during filling. In the consecutive process step, the fill volume is reduced by dosing. Thus, the powder is moved upwards by the lower punch, resulting in a rearrangement of the particles, which may lead to a change in the density of the bulk (PS4). Furthermore, at higher paddle speeds, additional powder may be above the die during dosing out, acting as a resistance against powder ejection and leading to densification as well (PS4). As MCC has a higher compressibility based on the Compressibility index compared to DCPA (Figure 5c), the increase in the filling yield due to the overfill for MCC is considerably higher compared to DCPA. Although for DCPA a densification with increasing paddle speed is also observable at a fill depth of 14 mm, the influence of the overfill on the filling yield is negligible.

**Figure 13:**
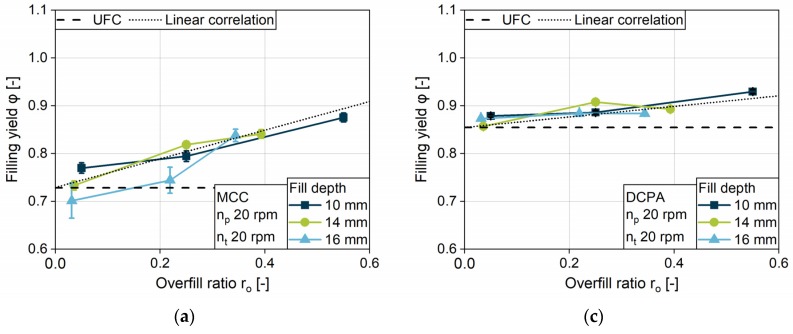

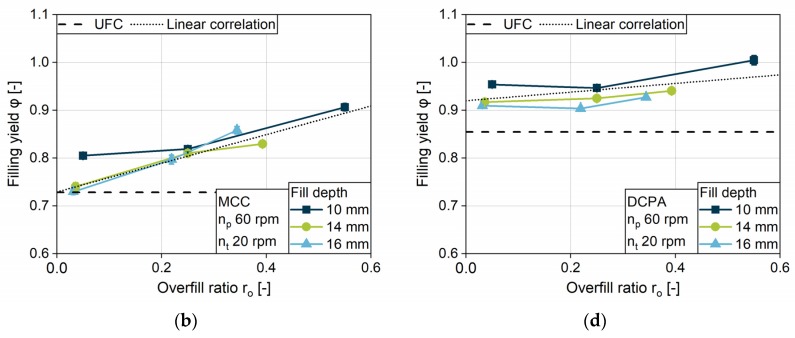
Filling yield at overfill ratios for the three fill depths at a turret speed of 20 rpm for MCC at a paddle speed of 20 (**a**) and 60 rpm (**b**) and, for DCPA, at a paddle speed of 20 (**c**) and 60 rpm (**d**).

In order to deeper evaluate densification due to the overfill ratio, filling yields for MCC and DCPA at every overfill ratio used are interpreted (Figure 13). At every fill depth, three different dosing depth were used, which led to three overfill ratios each. To simultaneously show the impact of the paddle speed, the filling yield is displayed for 20 rpm (Figure 13a,c) and 60 rpm (Figure 13b,d) paddle speed. It can be observed that, for both powders, MCC and DCPA, a linear correlation between the overfill ratio and the densification—the filling yield above *UCF*—is assumable, indicated by the dotted lines. The slope is determined by powder properties. More susceptible MCC displays a higher slope and, through that, a higher influence of overfill. For the investigated process parameters, the paddle speed has no considerable impact on the slope. Both powders show an increased filling yield for 60 rpm paddle speed at a fill depth of 10 mm even at low overfill ratios, which may result from the higher powder volume inside the feed frame due to the lower outlet powder flow. While for MCC, the filling yield at low overfill ratio is in accordance to the *UFC*, DCPA shows densification even at low overfill ratios at a paddle speed of 60 rpm. In comparison, at a low paddle speed of 20 rpm the filling yield of DCPA is in good accordance with the *UCF*, so the powder is not densified. Therefore, the densification of a powder with excellent flowability is more affected by the dynamic parameters like the paddle speed than by the overfill ratio. Besides the reduced interparticular forces for DCPA, the susceptibility to centrifugal acceleration is higher due to the higher true density compared to MCC. This might lead to an increased radial mass flow into the filling area, and thus to higher filling yields at low overfill ratios. In comparison, fairly flowing and compressible powders such as MCC present a stronger dependence on the overfill ratio for the increase in the apparent density inside the die during dosing out.

### 4.3. Impact of Material Properties on the Filling Yield

The formulation, and therefore the material properties, have a strong influence on die filling. As the flow behavior affects the powder movement inside the feed frame and the flow into the dies, the capability of particle rearrangement at low stresses has an impact on the densification of the powder. The investigation of the influence of the material properties on filling yield complements the analysis of the process parameters.

#### 4.3.1. Impact of the Material Properties on the Densification

Besides the challenge to fill the dies completely, there are process parameters which induce densification of the powder. Two different mechanisms are identified for densification: densification with increasing paddle speed and densification with increasing overfill ratio (Equation (3)).

**Figure 14:**
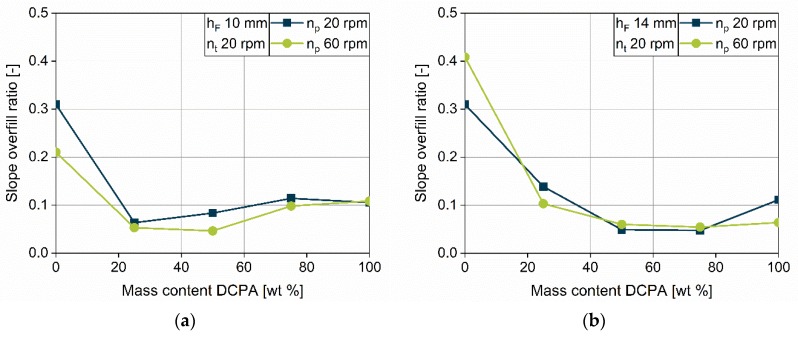

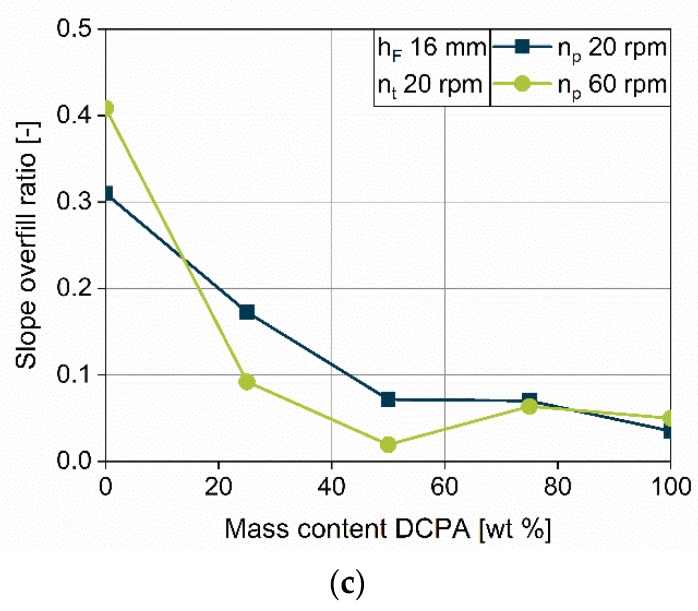
Slope of the linear fits of filling yield over the overfill ratio in dependence on the mass content of DCPA at a turret speed of 20 rpm at fill depth of 10 (**a**), 14 (**b**) and 16 mm (**c**).

**Figure 15:**
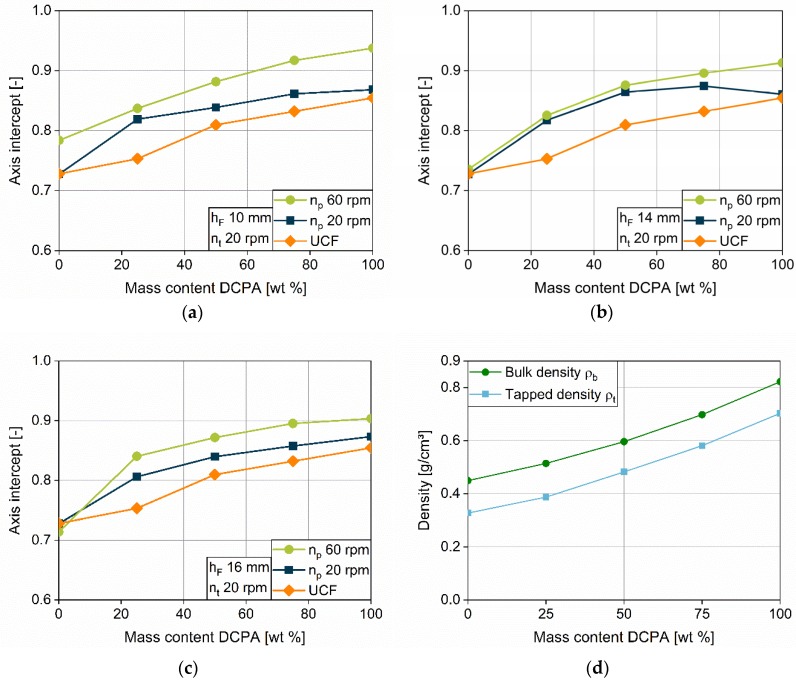
Axis intercept of the linear fits of the overfill ratio in dependence on the mass content of DCPA at a turret speed of 20 rpm at fill depths of 10 (**a**), 14 (**b**) and 16 mm (**c**); Bulk and tapped density of the five formulations used (**d**).

Densification due to the overfill ratio is presented in Section 4.2.6 with a focus on the impact of the process parameter. A strong dependence on material properties was already observed for DCPA and MCC. For further investigations, the filling yield, depending on the overfill ratio, is fitted with a linear equation for each fill depth and the slope, and the axis intercept are evaluated for the five formulations. While the slope characterizes the influence of the overfill ratio, the axis can help to identify the impact of different paddle speeds on the filling yield. The data are displayed over the mass content of DCPA (Figure 14 and Figure 15). As described in Section 4.2.6, the lowest turret speed of 20 rpm is used to ensure complete die filling and to exclude dynamic filling limitations (Figure 11a,d). To differentiate the impact of the paddle speed, data for 20 and 60 rpm are investigated.

For the slope, one can observe hyperbolic courses over the mass content of DCPA at both paddle speeds for all fill depths. MCC provides considerably high slopes, while the values decrease drastically with increasing DCPA content. A high positive slope indicates a strong impact of the overfill ratio on the apparent density inside the die. Unexpectedly, the slopes of the blends are almost as low as for DCPA, which does not correlate with the Compressibility index (Section 4.1). Therefore, another parameter, characterizing the reduction in density, is necessary to correlate this with the densification of the powder inside the die due to the overfill ratio.

For the two paddle speeds of 20 and 60 rpm, no considerable and systematic differences can be observed. The courses for fill depth of 14 and 16 mm are in good accordance, while, at 10 mm fill depth, the values for the slopes differ slightly. For MCC and the blend with 25 wt % DCPA, lower slopes are found, while the slopes for DCPA and the blends with a higher DCPA content are slightly higher. This indicates a higher impact of the overfill ratio at low fill depth. However, this can be related to the increased fill level inside the feed frame for free-flowing powders, which is assumed to be higher with lowering dosing depths. Thus, the higher slope might be due to an increased apparent density inside the die and probably in the feed frame during filling or because of the increased resistance against powder ejection during dosing.

The axis intercept, investigated at paddle speeds of 20 and 60 rpm, provides information about the theoretical apparent filling yield inside the die at an overfill ratio of zero, so no volume reduction takes place. Thus, the influence of other process parameters, as the paddle speed, on the densification is observable. Besides the data for the axis intercept at the paddle speeds of 20 and 60 rpm, the *UCF* is displayed to compare the data to the status of unconsolidated filling.

For the axis intercept of the five formulations, the data for a paddle speed of 20 rpm are below 60 rpm, which shows a higher densification for a higher paddle speed as expected. Furthermore, the difference between the intercepts of the two paddle speeds increases with increasing DCPA mass content. The difference reflects the stronger influence of the paddle speed on the apparent density inside the die, and probably inside the feed frame, for excellently flowing powders. It easily enters the die cavity during filling, and might firstly be densified by the high amount of powder provided by the paddles, which overtake the dies (PS3). The good flowability of these formulations allows fast and easy rearrangement of the particles, which can contribute to a denser bulk. Further densification takes place during dosing out (PS4). Even MCC shows a higher axis intercept for a paddle speed of 60 rpm at a fill depth of 10 mm, which might be explained by the higher fill level inside the feed frame at such a low fill depth.

In addition, the axis intercept at 20 rpm in comparison to the *UCF* is of special interest. For the pure substances MCC and DCPA, the intercepts are in particularly good agreement with the *UCF*s of the powders. Thus, at a paddle speed of 20 rpm the dies are completely filled at an apparent density equivalent to the bulk density without dosing out. Densification does not occur. In contrast to the results for the pure substances, the blends do not show a good agreement between the axis intercept and the *UCF*. The higher intercepts represent an apparent density higher than the bulk density of the blends. Thus, due to mixing effects, the blends show a different densification behavior in relation to the bulk density compared to the pure powders MCC and DCPA. A possible explanation can be found by regarding the bulk and tapped densities of the blends (Figure 15d). The bulk and tapped densities of the mixtures are below a theoretical linear mixing rule. This means that the bulk porosity of the combined pure substances with equivalent ratios is lower than after mixing them to a homogenous blend. The bulk porosity of the blends might increase due to interacting particle properties. Hypothetically, the higher axis intercept of the blends compared to the *UCF* can be explained by the reduction in the increased porosity under stress, which is applied at a paddle speed of 20 rpm.

#### 4.3.2. Impact of Flow Properties

**Figure 16:**
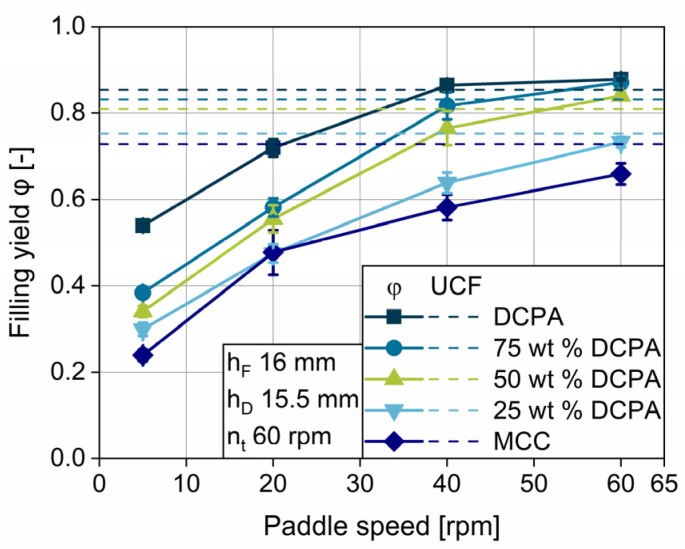
Impact of the paddle speed on the filling yield for the five formulations used at a turret speed of 60 rpm, a fill depth of 16 mm and a dosing depth of 15.5 mm.

The findings for the highly differing flow properties of pure MCC and DCPA are extended to the influence of the material properties on basis of the blends of these two. For the turret speed of 60 rpm, one can observe ranked graphs for the filling yields of the five formulations used (Figure 16), according to the ranking of the flow properties (Figure 5a,b). At a given paddle speed, DCPA has significantly higher values compared to the other formulations. DCPA first achieves complete filling, closely followed by the blend with 75 wt % DCPA. At a turret speed of 60 rpm, MCC and the blend with 25 wt % DCPA do not reach the *UCF*, owing to their considerably worse flow properties. The higher the mass content of MCC, the higher the minimum paddle speed to achieve complete filling. Therefore, the kinetic limitation is linked to the powder properties.

**Figure 17:**
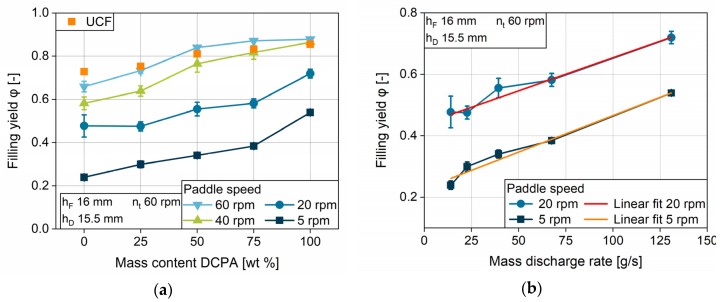
Impact of the mass content of DCPA on the filling yield (**a**), and correlation of the mass discharge rate from a hopper (25 mm orifice, Figure 5a) with the filling yield of the corresponding formulations at paddle speeds of 5 and 20 rpm (**b**).

In order to gain deeper understanding of the influence of the blend composition, the filling yield is presented in dependence on the mass content of DCPA (Figure 17a). At paddle speeds of 5 and 20 rpm, the filling yield for all formulations is far below the *UCF*, which is marked for each formulation by an orange dot. The course of the filling yield with increasing DCPA mass content is comparable to the one of the mass discharge rate (Figure 5a). To determine the relationship between the two parameters, they are plotted against each other (Figure 17b). The graphic shows a linear correlation between the filling yield of the five different formulations at paddle speeds of 5 and 20 rpm and the respective mass discharge rate. Therefore, at a specific paddle speed, the filling yield of different powders correlates with the mass discharge rate. An increasing paddle speed is described by a parallel shift, accounting for the provision of higher powder masses by the paddle feeder. 

The good correlation with the mass discharge rate might be due to the fact that the effect of suction filling is negligible at the used setting. Therefore, the filling of the dies is a similar process to flowing through an orifice, although air effects might differ as it needs to pass the flowing powder in case of die filling. However, it has to be considered that the diameter of the orifice used at the granular flow tester is 25 mm, while the dies have a diameter of 9 mm. Nevertheless, the correlation between the powder property and the filling yield of different powders enables first predictions of the filling yield that are below the *UCF* for different powders.

## 5. Conclusions

The presented work focuses on a systematic evaluation of the impact of process parameters and material properties on the result of die filling, expressed as the dimensionless filling yield. For an increase in turret speed, and therefore in tablet production rate, the dies can be completely filled only up to a critical velocity, dependent on the applied paddle speed and in particular on the material properties such as the flowability. Exceeding this velocity, the kinetic limitation is reached, and the filling yield declines approximately linearly. For fairly flowing powders (MCC), this critical turret speed is lower in comparison to good flowing formulations (DCPA). In addition, the tablet weight variation coefficient is considerably higher and rises with increasing turret speed. An increase in paddle speed shows a degressive rise in the filling yield for all powders presented and allows higher critical turret speeds, still achieving complete filling. The better the flowability, the lower the minimum paddle speed required to achieve die filling at a certain turret speed. Furthermore, higher paddle speeds lead to smaller variation coefficients. For low paddle speeds (5, 20 rpm) a limited powder mass, which entered the die as of a certain turret speed, is observed for DCPA and MCC, independent from the dosing depth.

Higher fill depth and therefore higher dosing depth increase the challenge of obtaining complete filling naturally, especially for fairly flowing powders (MCC). Besides the challenge of filling the dies completely, densification of the powder is observed, exceeding the *unconsolidated complete die filling*. Two mechanisms are identified which lead to denser bulks during filling: the paddle speed and the dosing out, quantified by the overfill ratio. With increasing paddle speed, especially at low turret speeds and a low dosing depth, the powder in front of the paddles and inside the dies can be densified up to the tapped density for good flowing powder (DCPA). Overfill ratio is identified as an independent factor that causes a linear increase in density. The analysis of the slope and axis intercept of the filling yield over the overfill ratio at different paddle speeds displayed the influence of the process parameters on densification, dependent on the material properties. While the overfill ratio is responsible for an increase in the apparent density for compressible powders such as MCC, the paddle speed has a considerable impact on the densification of excellent flowing powders as DCPA and blends with a high DCPA mass content. However, a correlation with measured powder properties as the Compressibility index was not identified. Besides the densification, the filling yield below *UCF* differs at the same process parameters for different materials. A linear correlation between the filling yield and the mass discharge rate from an orifice was found for low paddle speeds of 5 and 20 rpm.

Based on the general correlations identified in these findings, it is necessary to find appropriate process parameters to achieve complete filling without densifying the powder. Therefore, the paddle speed needs to be high for fairly flowing powders, while the overfill ratio should be small if densification is undesired. In contrast, for good flowing powders, the paddle speed should not be considerably higher than needed to fill the dies completely, if densification is to be avoided.

In the future, a prediction of the filling yield should be searched for. Therefore, the courses of the filling yield depending on the dynamic process parameters need to be mathematically described and the influence of the material properties needs to be quantified and correlated to measurable properties. The findings presented here deliver a basis for the model development. Moreover, a scale transfer to larger productions presses and different feeding systems is envisaged.

## Figures and Tables

**Table 1:** Characteristic particle sizes, solid, bulk and tapped density for microcrystalline cellulose (MCC) and anhydrous dicalcium phosphate (DCPA).

Material	x_10_ [µm]	x_50_ [µm]	x_90_ [µm]	ρ_s_[g/cm^3^]	ρ_b_[g/cm^3^]	ρ_t_[g/cm^3^]
MCC	28	110	243	1.56	0.327	0.449
25 wt % DCPA	29	117	258		0.387	0.514
50 wt % DCPA	31	136	284		0.483	0.596
75 wt % DCPA	31	148	298		0.581	0.698
DCPA	38	178	322	2.83	0.703	0.822

**Table 2:** Composition of the blends of MCC and DCPA processed on the XL 100.

Blend	wt % MCC	wt % DCPA + 1% MgSt
25 wt % DCPA	75	25
50 wt % DCPA	50	50
75 wt % DCPA	25	75

**Table 3:** Process parameters for tablet production with XL 100.

Fill Depth (mm)	Dosing Depth (mm)	Turret Speed (rpm)	Paddle Speed (rpm)
10	4.5 8.5 9.5	20, 40, 60	5, 20, 40, 60
14	8.5 10.5 13.5	20, 40, 60	5, 20, 40, 60
	10.5	20, 40, 60	5, 20, 40, 60
16	13.5		
	15.5	20, 30, 40, 50, 60	5, 20, 40, 60
